# Beyond traditional prognostics: integrating RAG-enhanced AtlasGPT and ChatGPT 4.0 into aneurysmal subarachnoid hemorrhage outcome prediction

**DOI:** 10.1007/s10143-025-03194-w

**Published:** 2025-01-11

**Authors:** Alim Emre Basaran, Agi Güresir, Hanna Knoch, Martin Vychopen, Erdem Güresir, Johannes Wach

**Affiliations:** Department of Neurosurgery, University Hospital Leipzig, Leipzig, Saxony Germany

**Keywords:** Aneurysmal subarachnoid hemorrhage, Artificial intelligence, AtlasGPT, ChatGPT, Neurologic outcomes, Prediction

## Abstract

To assess the predictive accuracy of advanced AI language models and established clinical scales in prognosticating outcomes for patients with aneurysmal subarachnoid hemorrhage (aSAH). This retrospective cohort study included 82 patients suffering from aSAH. We evaluated the predictive efficacy of AtlasGPT and ChatGPT 4.0 by examining the area under the curve (AUC), sensitivity, specificity, and Youden’s Index, in comparison to established clinical grading scales such as the World Federation of Neurological Surgeons (WFNS) scale, Simplified Endovascular Brain Edema Score (SEBES), and Fisher scale. This assessment focused on four endpoints: in-hospital mortality, need for decompressive hemicraniectomy, and functional outcomes at discharge and after 6-month follow-up. In-hospital mortality occurred in 22% of the cohort, and 34.1% required decompressive hemicraniectomy during treatment. At hospital discharge, 28% of patients exhibited a favorable outcome (mRS ≤ 2), which improved to 46.9% at the 6-month follow-up. Prognostication utilizing the WFNS grading scale for 30-day in-hospital survival revealed an AUC of 0.72 with 59.4% sensitivity and 83.3% specificity. AtlasGPT provided the highest diagnostic accuracy (AUC 0.80, 95% CI: 0.70–0.91) for predicting the need for decompressive hemicraniectomy, with 82.1% sensitivity and 77.8% specificity. Similarly, for discharge outcomes, the WFNS score and AtlasGPT demonstrated high prognostic values with AUCs of 0.74 and 0.75, respectively. Long-term functional outcome predictions were best indicated by the WFNS scale, with an AUC of 0.76. The study demonstrates the potential of integrating AI models such as AtlasGPT with clinical scales to enhance outcome prediction in aSAH patients. While established scales like WFNS remain reliable, AI language models show promise, particularly in predicting the necessity for surgical intervention and short-term functional outcomes. The study explored the use of advanced AI language models, AtlasGPT and ChatGPT 4.0, to predict outcomes for patients with aneurysmal subarachnoid hemorrhage (aSAH). It found that AtlasGPT provided the highest diagnostic accuracy for predicting the need for decompressive hemicraniectomy, outperforming traditional clinical scales, while both AI models showed promise in enhancing outcome predictions when integrated with established clinical assessment tools.

**Supplementary Information:**

The online version contains supplementary material available at 10.1007/s10143-025-03194-w.

## Introduction

Aneurysmal subarachnoid hemorrhage (aSAH) is a severe medical condition with high pre- and in-hospital mortality rates [1–3]. Clinical outcomes depend mainly on the initial severity of aSAH [4–6]. In patients with intractably elevated intracranial pressure (ICP), decompressive craniectomy (DC) may be indicated as a life-saving procedure [7].

Predicting the prognosis of aSAH patients is a challenging task. Machine learning algorithms have the potential to make prognostic predictions based on individual patient parameters [8, 9]. A pilot study has shown that large language models or Generative Pre-trained Transformers (GPT) such as ChatGPT, have the potential to predict short-term functional outcomes in acute ischemic stroke patients after thrombectomy more accurately than the existing risk scores [10]. However, it should be noted that the large language models used in the studies have not yet been explicitly developed for medical purposes. Atlas GPT was specifically developed and trained for neurosurgical questions, enabling more precise answers to complex questions and potentially improving prognostic performance [11].

To our knowledge, no study has yet investigated the use of large language models in relation to aSAH. However, based on the few studies conducted so far in other medical fields, there is promising potential. Against this backdrop, we compared the prognostic accuracy of the large language models ChatGPT 4.0 and AtlasGPT based on individual aSAH patient parameters for predicting in-hospital mortality, neurological outcome, and the need for DC.

## Methods

### Study setting & participants

A retrospective analysis was conducted on patients diagnosed with aSAH at the Department of Neurosurgery at Leipzig University Hospital. The study included patients treated according to corresponding guidelines between 2019 and 2022.

### Ethics

The retrospective study was conducted in compliance with the Declaration of Helsinki and its amendments and was approved by the local medical ethics committee (387/23).

### Data collection

Retrospective data was collected from patient records and ICU reports for the study. The following clinical, radiological, and laboratory data were recorded: Baseline demographic patient characteristics (Age, sex), Word Federation of Neurological Surgeons (WFNS) Score, Glasgow Coma Scale (GCS) score, pupillary reflex, neuroradiological parameters (Fisher score, intracerebral hemorrhage, midline shift, location of ruptured aneurysm, presence of initial hydrocephalus, Subarachnoid Hemorrhage Early Brain Edema Score (SEBES)) surgical treatment modalities (clipping, endovascular coiling), and laboratory values (hemoglobin, CRP, blood lactate, serum creatinine) [12, 13].

### Outcomes

The study’s primary outcome was in-hospital survival. The secondary outcome was favorable neurological outcome, which was based on the modified Rankin Scale (mRS) and defined as a mRS score of 0–2 [14]. Tertiary outcome parameter was the need for DC.

## Development of a chat prompt for Atlas GPT and ChatGPT 4.0

In the creation of a standardized dialogue prompt, our methodology adopted an iterative process, as recommended by Kanjee et al. [15]. An initial text was composed and subsequently refined through a process of trial and error, with the aim of eliciting specific responses from ChatGPT-4 and AtlasGPT. This initial text comprehensively described the task and context to the language model. The full standardized dialogue prompt is accessible (see supplementary file 1). In summary, the language model was instructed to assume the role of an “AI intensive care physician” or an “AI neurosurgeon” tasked with managing a patient who was admitted on hospital for aSAH and aneurysms were treated via Coiling or Clipping within 24 h after the aSAH event. Additionally, the model was provided with seventeen baseline patient-, disease-, and procedure-specific parameters, chosen for their recognized value in predicting outcomes after aSAH. This standardized anonymized way was chosen because the unstructured upload of medical records to a cloud-based language model would result in significant data privacy concerns. The text was developed in a standardized way until AtlasGPT (Atlasmeditech LLC. 2024) and ChatGPT 4.0 (OpenAI, Inc., San Francisco, USA) could answer questions with a simple ‘yes’ or ‘no’. The following baseline factors of aSAH at admission were considered in the analysis: WFNS grade, pupillary reflex, age, gender, Glasgow coma scale, Fisher scale, intracerebral hemorrhage, intraventricular hemorrhage, midline-shift, location of ruptured aneurysm, treatment modality, medical treatment with antiplatelet drugs after endovascular treatment, initial hydrocephalus treatment such as external ventricular drain, hemoglobin, CRP, blood lactate, and serum creatinine. AtlasGPT and ChatGPT 4.0 were asked four questions that required ‘yes’ or ‘no’ answers after entering the text and parameters (see supplementary file 1).

1. Will this patient survive to hospital discharge? Please provide a yes/no answer.
2. Will this patient experience a good neurological outcome at hospital discharge as defined by the modified ranking scale (0–2). Please provide a yes/no answer.
3. Will this patient experience a good neurological outcome at 6-months after aneurysmal subarachnoid hemorrhage e as defined by the modified ranking scale (0–2). Please provide a yes/no answer. “.
4. Will the patient have to be treated by a decompressive craniectomy within the next week? Please provide a yes/no answer.

The responses were recorded in an Excel file. Each question was asked three times using both AtlasGPT and ChatGPT 4.0. A new chat was opened for each question. In the case of dichotomous answers (yes/no), the most frequent response from the repeated questions was considered. For example, if the responses were yes/yes/no, the overall response was recorded as ‘yes’. In cases where ‘no’ was the more frequent response, we selected ‘no’ as the final answer. If we did not receive a response to a question, we reopened the chat and repeated the question until we received a complete and unambiguous answer. We used only complete and unambiguous answers for statistical analysis. The chat prompts were performed between the 5th and 30th of March in 2024.

### Statistical analysis

Receiver operating characteristics curves (ROC) curves and area under curves (AUC) values were calculated to determine the prognostic significance of AtlasGPT, ChatGPT 4.0, WFNS grading, Fisher scale, and SEBES. Median values and interquartile ranges (IQR) of metric data are reported. The prognostic significance of those parameters were determined by sensitivity, specificity, cut-off values, positive/negative predictive value, and the Youden’s index. Statistical analyses was performed using SPSS Statistics version 29.0.2.0 (IBM, Armonk, New York). Statistical graphics were created using SPSS version 29.02.0 and were modified with corresponding tables using BioRender.com.

## Results

### Study cohort

**Fig. 1:**
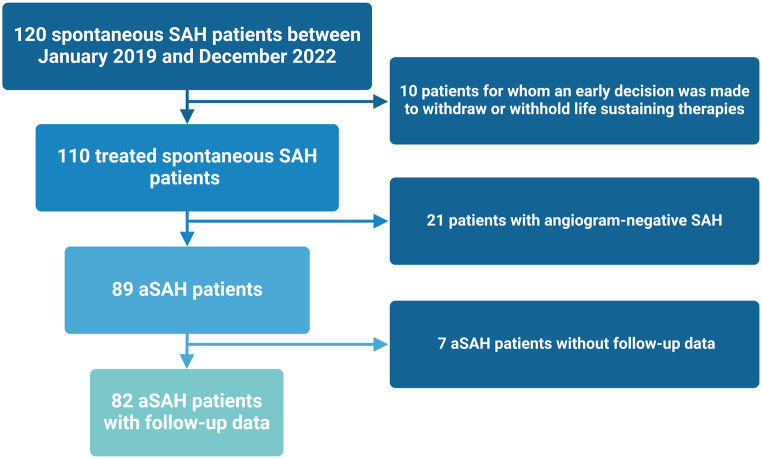
Study flowchart over 48 months with a total study cohort of 82 consecutive patients

Between January 2019, and December 2022, 120 spontaneous aSAH patients were admitted to the present institution. After the exclusion of patients with an withdraw or withhold from life sustaining therapies, patients with an angiogram-negative SAH, and 7 patients who were lost to follow-up, 82 consecutive aSAH patients were included in the present investigation. Figure 1 summarizes the process flow chart.

### Patient characteristics

The cohort comprised predominantly middle-aged individuals with a median age of 56 years (IQR 44.8–67.3), with a slight female predominance (49/82; 59.8%). The majority of patients presented with poor-grade subarachnoid hemorrhage, reflected by WFNS gradings IV and IV in 50 patients (50/82; 61.0%). A considerable number exhibited signs of brain edema on imaging as evidenced by SEBES scores, with 19 patients (23.2%) being assigned the highest score (IV). Baseline vigilance was generally compromised, indicated by a median Glasgow Coma Scale score of 7 (IQR 3.0–14.0). Intracranial pathology was confirmed by the presence of midline shift in 24 patients (29.3%). The aneurysms were most frequently located at the anterior cerebral artery (ACA) complex (36/82; 43.9%) and the middle cerebral artery (MCA) complex (25/82; 30.5%). Most patients underwent endovascular treatment for their aneurysms (54/82; 65.9%). Further patient- and disease-specific characteristics are summarized in Table 1.

**Table 1:** Patient characteristics

Characteristic	Frequency (*n* = 82)
Age, Median (IQR)	56.0 (44.8–67.3)
Sex	
	49 (59.8%)
Male	33 (40.2%)
1	18 (22.0%)
2	9 (11.0%)
3	5 (6.1%)
4	9 (11.0%)
5	41 (50.0%)
Fisher scale	
1	1 (1.2%)
2	3 (3.7%)
3	62 (75.6%)
4	16 (19.5%)
SEBES	
0	16 (19.5%)
1	12 (14.6%)
2	20 (24.4%)
3	15 (18.3%)
4	19 (23.2%)
GCS, Median (IQR)	7.0 (3.0–14.0)
Midline-shift	
Present	24 (29.3%)
Aneurysm location	
Anterior complex	
ACA	1 (1.2%)
Pericallosal artery	5 (6.1%)
AcoA	30 (36.6%)
ICA	
Bifurcation	4 (4.9%)
Ophtalmic artery	1 (1.2%)
AChoA	1 (1.2%)
PcoA	3 (3.7%)
MCA	
M1	5 (6.1%)
Bifurcation	17 (20.7%)
M2	3 (3.7%)
Posterior circulation	
Basilar artery	6 (7.3%)
Vertebral artery	2 (2.4%)
PICA	3 (3.7%)
PCA	1 (1.2%)
Arterial hypertension	54 (65.9%)
Antiplatelet therapy	7 (8.5%)
Anticoagulation intake	3 (3.7%)
Baseline hydrocephalus (EVD placement)	45 (54.9%)
Type of aneurysm treatment	
Clipping	28 (34.1%)
Endovascular	54 (65.9%)
Lactate (mmol/I), Median (IQR)	1.6 (1.0-2.5)
C-reactive protein (mg/I), Median (IQR)	2.3 (1.1–4.5)
Serum creatinine (µmol/I), Median (IQR)	68.0 (57.0-85.5)
Hemoglobin (mmol/I), Median (IQR)	8.2 (7.4–8.7)

Abbrevations: IQR = Interquartile range; SEBES = subarachnoid hemorrhage early brain edema score; EVD = external ventricular drain; ACA = anterior cerebral artery; AcoA = anterior communicating artery; ICA = internal carotid artery; AChoA = anterior choroidal artery; MCA = middle cerebral artery; PICA = posterior inferior cerebellar artery; PCA = posterior cerebral artery

### Outcome parameters

During the hospitalization period, 22% of patients (*n* = 18) expired. DC was performed in 34.1% (*n* = 28) during hospital therapy. Primary decompressive hemicraniectomy was performed in 15 cases during surgical clipping (15/28; 53.6%). The residual decompressive hemicraniectomies were performed secondary due to intracerebral pressure elevations after surgery because of subdural-, epidural- and intracerebral hematomas (4/28; 14.3%), brain edema without signs of infarction (4/28; 14.3%), and radiologically determined infarction (5/28; 17.9%). At the time of hospital discharge, a favorable prognosis, denoted by a modified Rankin Scale score of ≤ 2, was observed in 28% of patients (*n* = 23). At the 6-month follow-up, an improvement in outcomes was noted; 46.9% of the survivors (*n* = 30) achieved a favorable functional status. Supplementary table S1 summarizes the outcome parameters, which were requested from the artificial intelligence language models.

### Prognostication

#### In-hospital survival

**Fig. 2:**
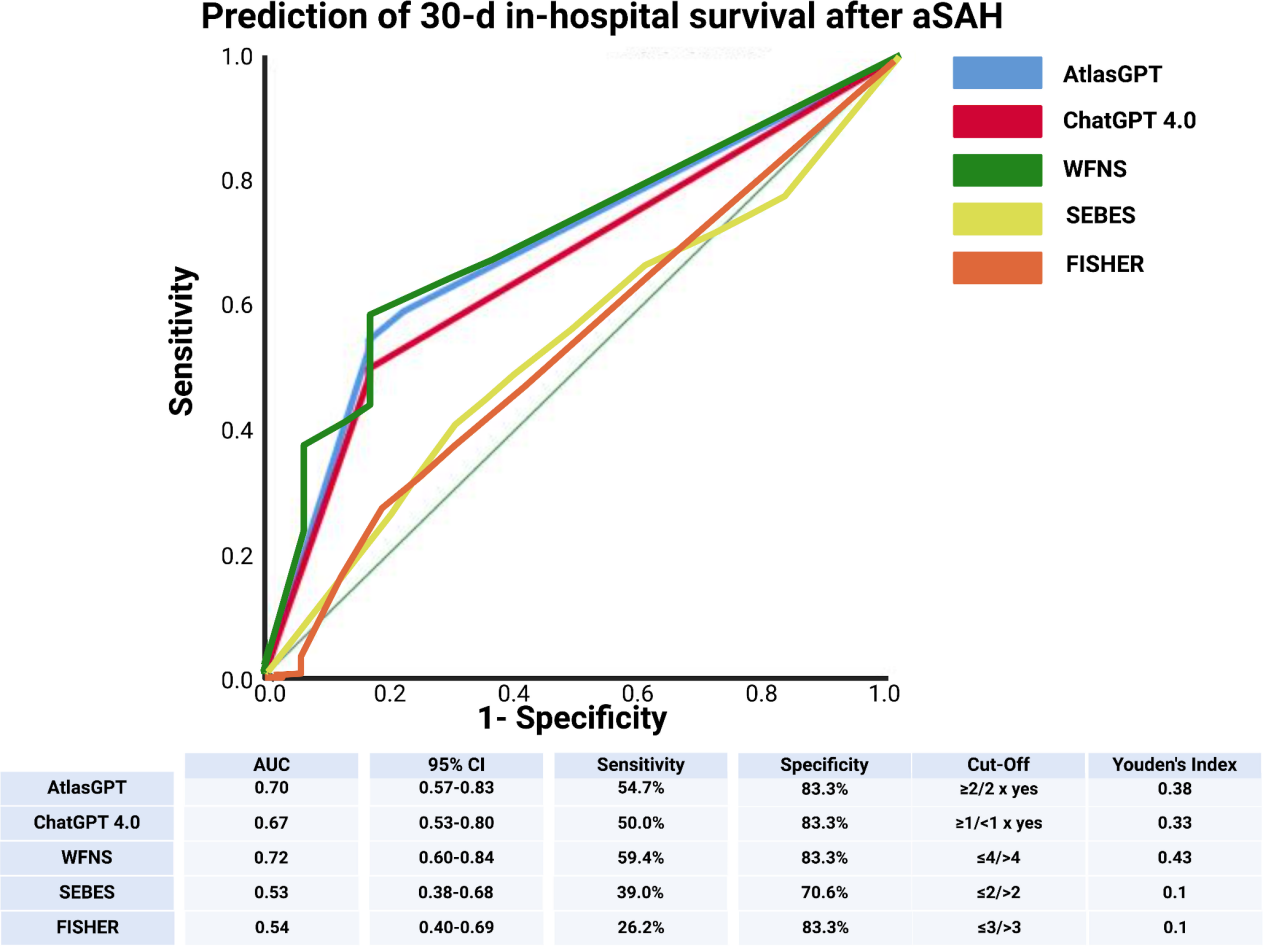
Comparison of ROC curves for in-hospital survival. Abbreviations include AUC: Area under the Curve, CI: Confidence Interval, GPT: Generative Pre-Trained Transformer, mRS: Modified Rankin Scale

For the endpoint of 30-day in-hospital survival, the World Federation of Neurological Surgeons (WFNS) grading scale manifested the superior discriminative capability with an AUC of 0.72 (95% CI: 0.60–0.84), coupled with a sensitivity of 59.4% and specificity of 83.3% with a cut off set at ≤ 4/>4. The positive predictive value and negative predictive values were 92.7% and 36.6%, respectively (see supplementary table S2). The WFNS scale’s Youden’s Index was 0.43, indicating a robust balance between sensitivity and specificity in this acute prognostic scenario at admission. Although AtlasGPT had a slightly lower AUC, it demonstrated a comparable performance in terms of specificity (83.3%) when responding that the patient will survive for at least two times of three chat prompt runs. AtlasGPT and ChatGPT 4.0 showed similar capabilities with AUCs of 0.70 (95% CI: 0.57–0.83) and 0.67 (95% CI: 0.53–0.80) respectively, while SEBES and FISHER scales demonstrated lower discriminative power with AUCs of 0.53 (95% CI: 0.38–0.68) and 0.54 (95% CI: 0.40–0.69). Figure 2 illustrates the findings.

### Need for decompressive hemicraniectomy during hospital therapy

**Fig. 3:**
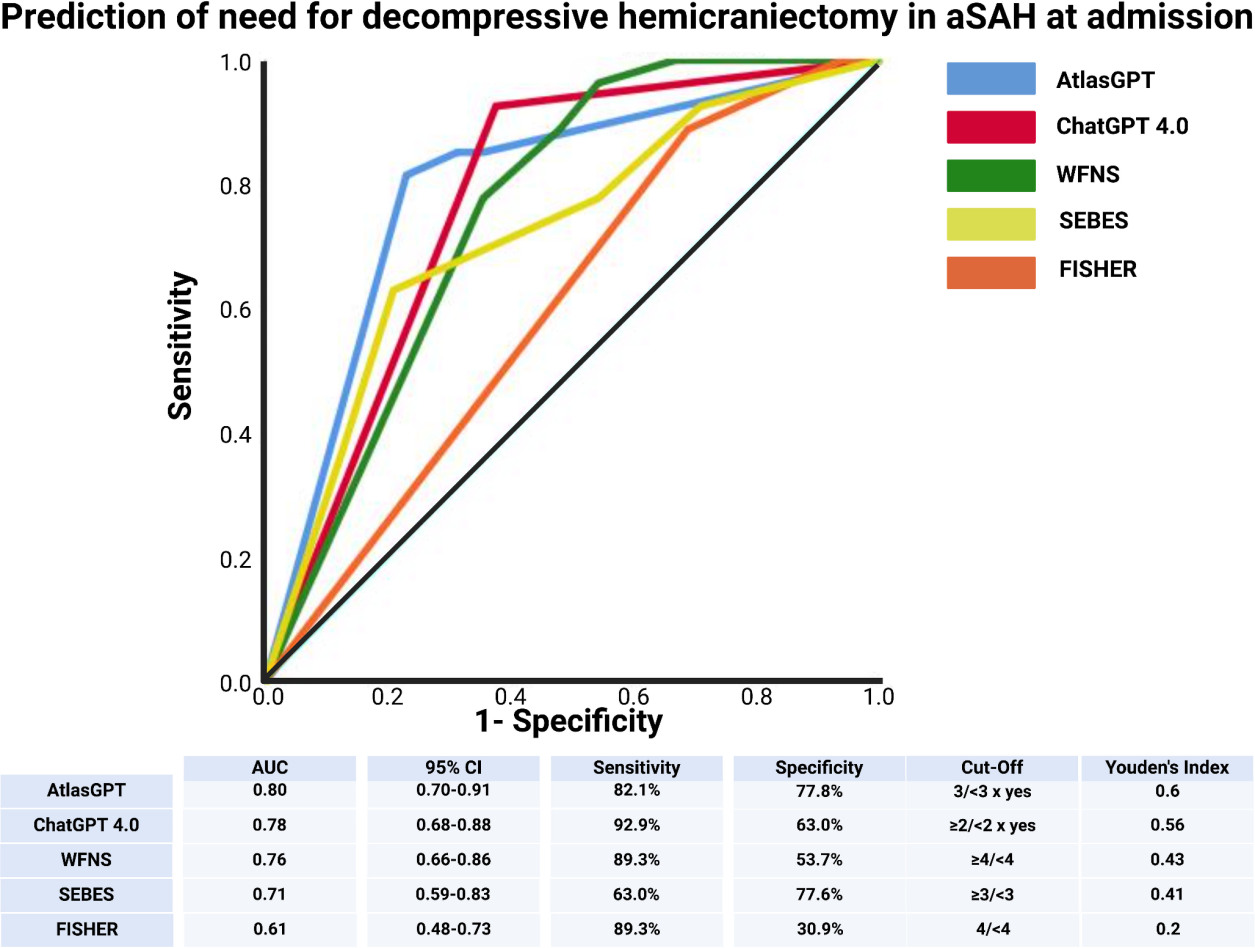
Comparison of ROC curves for need for decompressive hemicraniectomy. Abbreviations include AUC: Area under the Curve, CI: Confidence Interval, GPT: Generative Pre-Trained Transformer, mRS: Modified Rankin Scale

AtlasGPT presented the most pronounced diagnostic accuracy in anticipating the requirement for decompressive hemicraniectomy upon admission with an AUC of 0.80 (95% CI: 0.70–0.91). The model’s sensitivity reached 82.1%, with specificity at 77.8%, and a Youden’s Index of 0.6, when prognosticating the need for decompressive hemicraniectomy in three dialog runs in a row. The positive predictive value and negative predictive values of AtlasGPT indicating the need for decompressive hemicraniectomy were 65.7% and 89.4%, respectively (see supplementary Table 2). AtlasGPT´s predictive power was followed by ChatGPT 4.0, which had an AUC of 0.78 (95% CI: 0.68–0.88), and the WFNS score with an AUC of 0.76 (95% CI: 0.66–0.86). SEBES and FISHER scales had the lowest AUCs, indicating less predictive value for this outcome. Figure 3 summarizes the results of this analysis.

### Favorable outcome (mRS ≤ 2) at discharge

**Fig. 4:**
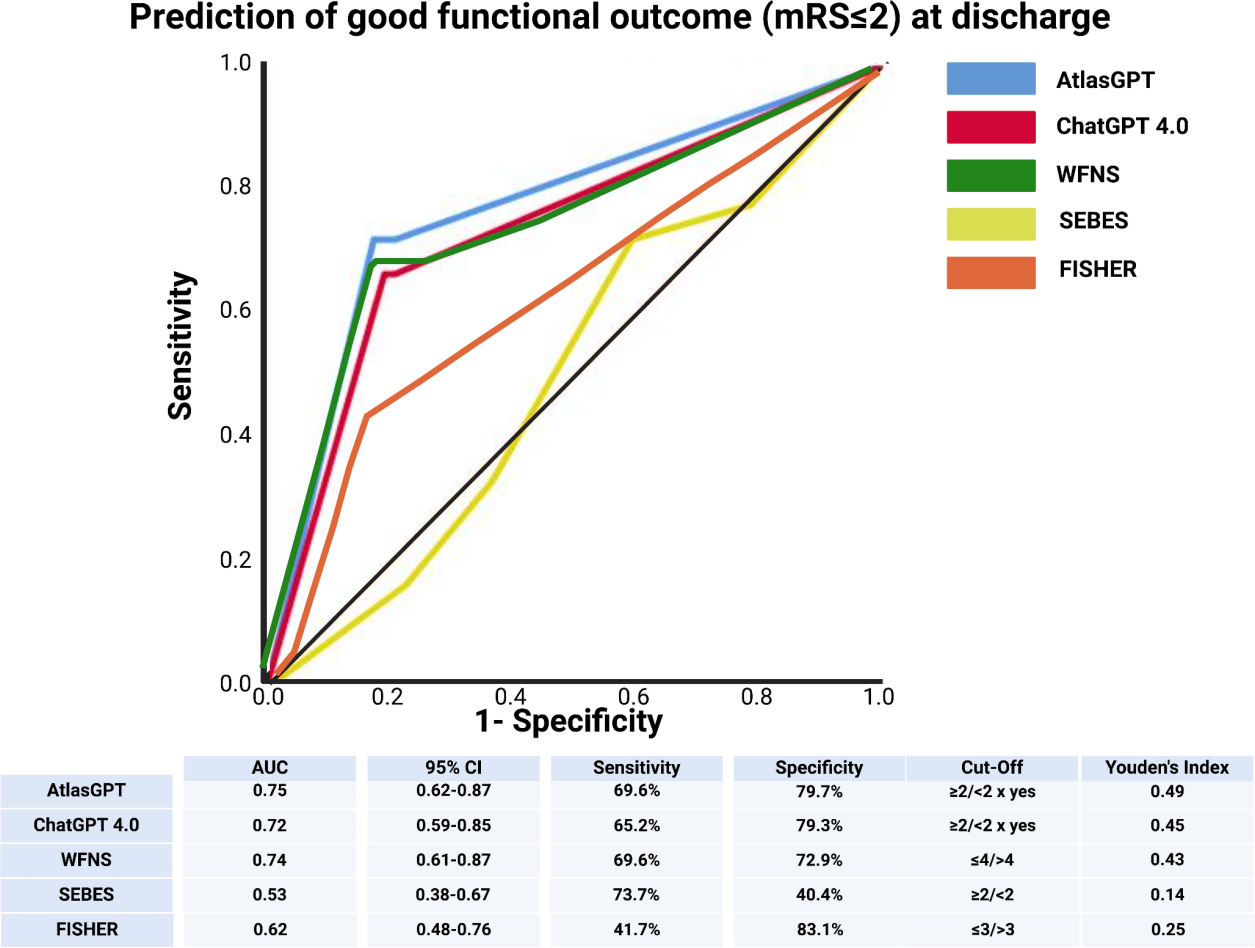
Comparison of ROC curves for favorable functional outcome (mRS ≤ 2) at discharge. Abbreviations include AUC: Area under the Curve, CI: Confidence Interval, GPT: Generative Pre-Trained Transformer, mRS: Modified Rankin Scale

The WFNS score and AtlasGPT both provided high prognostic value for predicting a good functional outcome at discharge, with AUCs of 0.74 (95% CI: 0.61–0.87) and 0.75 (95% CI: 0.62–0.87), respectively. The sensitivity and specificity of AtlasGPT were 69.6% and 79.7%, respectively, with an optimal cutoff at ≥ 2/<2 yes and a Youden’s Index of 0.49, suggesting good prognostic performance at the point of discharge. The positive predictive value and negative predictive values of AtlasGPT prognosticating favorable mRS at discharge were 57.1% and 87.0%, respectively (see supplementary Table 1). ChatGPT 4.0 also performed adequately with an AUC of 0.72 (95% CI: 0.59–0.85). The SEBES and FISHER scales were less predictive to assess clinical outcome, as reflected by their AUCs of 0.53 (95% CI: 0.38–0.67) and 0.62 (95% CI: 0.48–0.76). Figure 4 displays the results of the analyses regarding functional outcome at discharge.

### Favorable outcome (mRS ≤ 2) at 6-months

For the prediction of a favorable functional outcome at a 6-month interval, the WFNS grading scale was pre-eminent, delivering an AUC of 0.76 (95% CI: 0.64–0.88), with a sensitivity of 76.7% and specificity of 71.9%. The calculated Youden’s Index was 0.49, indicating a balanced predictive performance for long-term recovery assessments. The positive predictive value and negative predictive values of WFNS grading to prognosticate favorable mRS outcome at 6-months were 71.9% and 76.7%, respectively (see supplementary Table 2). AtlasGPT and ChatGPT 4.0 provided both comparable AUCs of 0.69 (95% CI: 0.56–0.83). SEBES and FISHER scores were less effective in predicting long-term outcomes, with AUCs of 0.58 (95% CI: 0.43–0.73) and 0.58 (95% CI: 0.44–0.72), respectively. Supplementary file 2 outlines the results.

### Response variability of language models concerning the prediction of endpoints

During each of the three trials involving AtlasGPT and ChatGPT 4.0, we encountered occurrences of divergent outputs, classified as response variability in response to the prompts given. AtlasGPT delivered different responses for the endpoints In-hospital mortality, need for decompressive hemicraniectomy, favorable outcomes at discharge, and favorable outcomes at 6-months after aSAH in 5 (6.1%), 7 (8.5%), 3 (3.7%), and 3 (3.7%) runs, respectively. ChatGPT 4.0 likewise generated diverse outcomes for in-hospital mortality, favorable discharge outcomes, and favorable 6-month outcomes post-aSAH, each occurring in 1 (1.2%) instance. No divergent responses for the necessity of decompressive hemicraniectomy was observed using ChatGPT 4.0.

## Discussion

The present study compared the prognostic value of the retrieval-augmented generation techniques enhanced large language model AtlasGPT based on peer-reviewed, reliable neurosurgery-specific evidence, the large language model ChatGPT-4.0 with well-validated clinical and imaging scales. The prognostic performance of AtlasGPT to predict the functional outcome at discharge and the need for decompressive hemicraniectomy in aSAH based on characteristics at hospital admission demonstrated substantial roles for artificial intelligence in clinical practice (see supplementary file 3). This finding is also supported by other studies showing that AI models are capable of accurately predicting specific interventions and the necessity for certain measures in emergency medicine and neurosurgery [8, 16]. Such applications could be particularly valuable in quickly identifying high-risk patients and enhancing decision making in emergency situations. Nevertheless, these results necessitate extensive discussion.

Both language models showed response variabilities, with slightly more variability in the AtlasGPT language model. However, we ran three iterations of four binary questions for 82 patients in two language models and observed only 21 instances of response variability (21/656; 3.2%). To address this issue seriously and reduce potential bias caused by response variability, we ultimately took the mean value of the responses for statistical processing. Nevertheless, it has to be noted that our investigation focused on the most advanced ChatGPT version currently available, which requires a paid subscription (namely, the GPT-4-based model). The freely accessible and more commonly used version is based on the 3.5 model, which might produce less reliable predictions with more response variability and temporal instability [17]. This demonstrates that artificial intelligence can be most effective when integrated with “human intelligence”, such as the expertise of a supervising clinician. Therefore, the present study suggests that a well-trained model like AtlasGPT might facilitate identifying high-risk for therapy refractory increased ICP. The current study represents only an initial step in this direction and demonstrates how specific training data for aSAH patients could further enhance model performance in neurosurgery [11]. Additionally, it highlights the necessity for users to closely monitor the application of large language models in clinical settings. Decisions about treatment limitations and prognosis are inherently challenging, demanding human attributes such as long-term professional expertise, empathy, and emotional insight. In contrast, large language models are purely machines operating on stochastic processes, lacking any form of consciousness or emotional capacity [18].

While the use of large language models in the medical field is growing, neurovascular research on their ability to predict patient outcomes remains limited. A retrospective analysis of clinical, neuroimaging, and procedure-related data involving 163 patients with acute ischemic stroke found that ChatGPT adequately predicted short-term functional mRS outcomes at 3-months after mechanical thrombectomy and was superior to existing risk scores [19].

Mortality and functional prognosis in aSAH are complex, with scales like Hunt and Hess and WFNS gauging risk based on initial assessments and traits but ignoring the effects of ending life support, which might bias outcomes [20]. Furthermore, there is growing concern that pessimistic views on severely injured patient´s recoveries might worsen outcomes, termed a “self-fulfilling prophecy” [21, 22]. However, it is of paramount importance to scientifically validate these language models because patients and their relatives will potentially start putting trust in what conventional AI, like ChatGPT, predicts. Despite language model designers taking steps to prevent these consultations by making sure that language models are not physicians and cannot give medical advices, such protocols can be bypassed by “jailbreak“ prompts, such as feigned scenarios as demonstrated in the present study [23].

Physicians evaluating large language models should also be mindful of the “stochastic parrot” concept [24, 25]. This principle highlights that due to their algorithmic nature, large language models lack comprehension of both the information they receive and the responses the produce, merely replicating learned patterns and biases, including stereotypes and social disparities [26]. These mechanisms might account for why the large language model´s performances in survival prediction and long-term neurological outcome prediction are comparable to, and not superior to, established clinical grading systems. This observation is consistent with findings from other research indicating similar levels of effectiveness in clinical or theoretical settings [27, 28]. However, we found a superior role of a generated dialog describing baseline patient, disease, and procedure-specific aSAH patient characteristics, which might facilitate the identification of those patients who will potentially undergo decompressive hemicraniectomy. Brain swelling and elevated ICP are established factors worsening outcomes after aSAH [29]. Despite decompressive hemicraniectomy being a well-established procedure with high-level evidence for space-occupying stroke, the currently used language models and including AtlasGPT, still have to rely on retrospective data. Further insights on the role of primary decompressive hemicraniectomy in aSAH and which patient will potentially benefit from this procedure might inform the randomized controlled PICASSO trial investigating decompressive hemicraniectomy in poor-grade (WFNS IV&V) aSAH patients [30].

### Limitations

To our knowledge, this is the first investigation using large language models for predicting outcomes following aSAH with real-world patient data. It adopts a practical method focused on reproducibility and the integrity of data. Nonetheless, this study is not without its drawbacks.

First, there is the tendency of large language models to produce response variability which have to be addressed by repetitive approaches and trusting the mean values. Additionally, ChatGPT 4.0 was not specifically developed for medical use compared to AtlasGPT. Hence, the effectiveness and accuracy of ChatGPT 4.0 in addressing clinical issues has yet to be established.

Each hospital has unique experiences with decompressive hemicraniectomy, and each neurosurgical department has its own perspectives on the procedure. This variability in clinical practice can influence patient outcomes and perceived effectiveness of the procedure. Some departments may have more favorable outcomes due to higher surgical volumes, specialized training, or different postoperative care protocols, while others might be more conservative in their approach. These differences can lead to varying degrees of success and differing opinions on the utility of decompressive hemicraniectomy, which can impact the generalizability of study results. Furthermore, every neurosurgical department´s unique experiences and opinions could be a limitation for this study, as these subjective factors are not controlled for and can introduce bias.

Finally, the present study represents a single-center cohort, which may limit their applicability to other settings or regions, underscoring the need for further studies across various environments to confirm the universality of these results. Finally, the present results have not been compared to a blinded experienced senior physician estimating the outcomes of the individual patients with the same baseline data. Therefore, the present study represents an initial step in the application and further development of these technologies in neurosurgery and demonstrates a clear perspective on how LLM systems might be applicated as a supplementary tools in. Further validation in large scale studies is needed.

## Conclusions

AtlasGPT demonstrated a good performance in predicting functional outcome at discharge and the need for decompressive hemicraniectomy and thus may be a helpful tool for early endpoints in risk predictions of aSAH patients. While AI models may not yet present a “gold standard” for clinical prognosis, their increasing accuracy and potential might offer a clear perspective for future applications. Recent studies in stroke prognosis and emergency medicine confirm that AI models can already provide valuable clinical insights, especially when used alongside human expertise [9, 16]. The present study demonstrates that AtlasGPT can already provide a good discrimination for the need of interventions (e.g., decompressive craniectomy for increased ICP) and highlights the need for further studies assessing this potential. Future research should focus on specific trained large language models and further validate various clinical endpoints.

## Electronic supplementary material

Below is the link to the electronic supplementary material.

Supplementary Material 1

Supplementary Material 2

Supplementary Material 3

Supplementary Material 4

## Data Availability

No datasets were generated or analysed during the current study.
