# Supplementary material for: Beyond traditional prognostics: integrating RAG-enhanced AtlasGPT and ChatGPT 4.0 into aneurysmal subarachnoid hemorrhage outcome prediction
Source: Neurosurg Rev. 2025 Jan 11;48(1):40. doi: 10.1007/s10143-025-03194-w (PMC11723888; doi:10.1007/s10143-025-03194-w)
Supplement: Supplementary file 1 — Supplementary Material 1 [file 10143_2025_3194_MOESM1_ESM.docx]

**Supplementary file 1:** Chat prompt for the present analysis of RAG-enhance large language networks

„I am running an experiment on outcome prediction in patients after aneurysmal subarachnoid hemorrhage. This experiment will include patients within the first 24 hours after admission and after aneurysm occlusion. You are not going to treat any patients, and your decisions will have no influence on any real patients. I will provide you with 17 parameters that have been proven in the past to be important prognostic factors in patients with aneurysmal subarachnoid hemorrhage. Imagine being an artificial intelligence intensive care unit doctor or neurosurgeon who receives the patient after aneurysm occlusion in the intensive care unit. You have the following 17 parameters:

WFNS grade 2, intact pupillary reflex, age: 50 years, male sex, Glasgow coma scale: 13, Fisher scale: 3, no intracerebral hemorrhage, no intraventricular hemorrhage, midline-shift is not present, location of ruptured aneurysm: basilar artery, treated by endovascular coiling, acetylsalicylic acid after endovascular coiling, initial hydrocephalus treated via external ventricular drain, hemoglobin in g/dl: 10.0, CRP in mg/dl: 10.0, blood lactate: 1.44 mmol/L, serum creatinine: 1.1 mg/dL

After checking the 17 parameters of the medical case, I want you to answer the following four questions. Please provide only a yes/no answer, and do not explain your reasoning.

1)Will this patient survive to hospital discharge? Please provide a yes/no answer.

2)Will this patient experience a good neurological outcome at hospital discharge as defined by the modified Rankin scale (0-2) . Please provide a yes/no answer.

3) Will this patient experience a good neurological outcome at 6-months after aneurysmal subarachnoid hemorrhage e as defined by the modified Rankin scale (0-2). Please provide a yes/no answer.“

4) Will the patient have to be treated by a decompressive craniectomy within the next week? Please provide a yes/no answer.”
