# Supplementary figures and images for: Beyond traditional prognostics: integrating RAG-enhanced AtlasGPT and ChatGPT 4.0 into aneurysmal subarachnoid hemorrhage outcome prediction
Source: Neurosurg Rev. 2025 Jan 11;48(1):40. doi: 10.1007/s10143-025-03194-w (PMC11723888; doi:10.1007/s10143-025-03194-w)

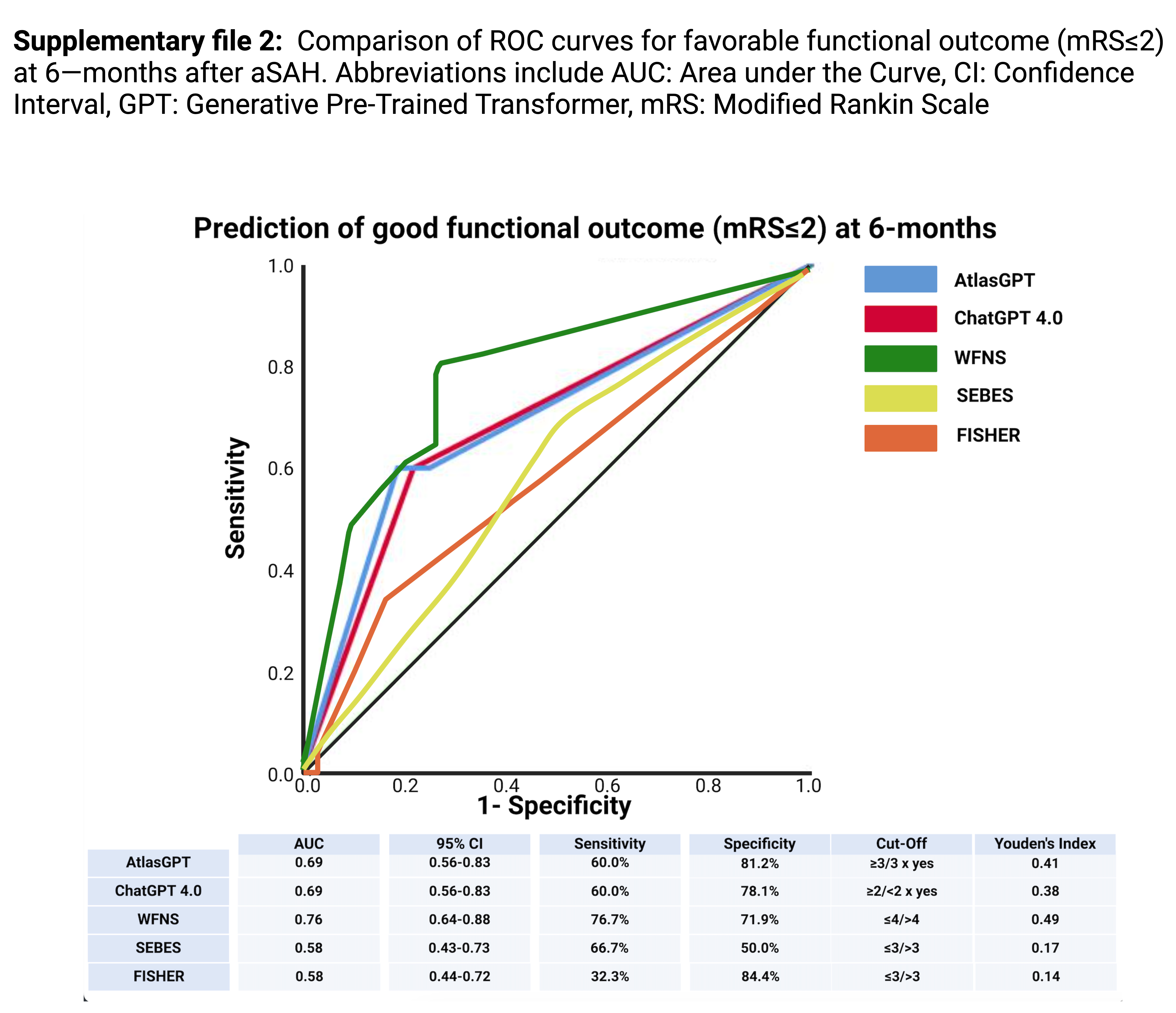

Supplement: Supplementary file 2 — Supplementary Material 2 [file 10143_2025_3194_MOESM2_ESM.png]

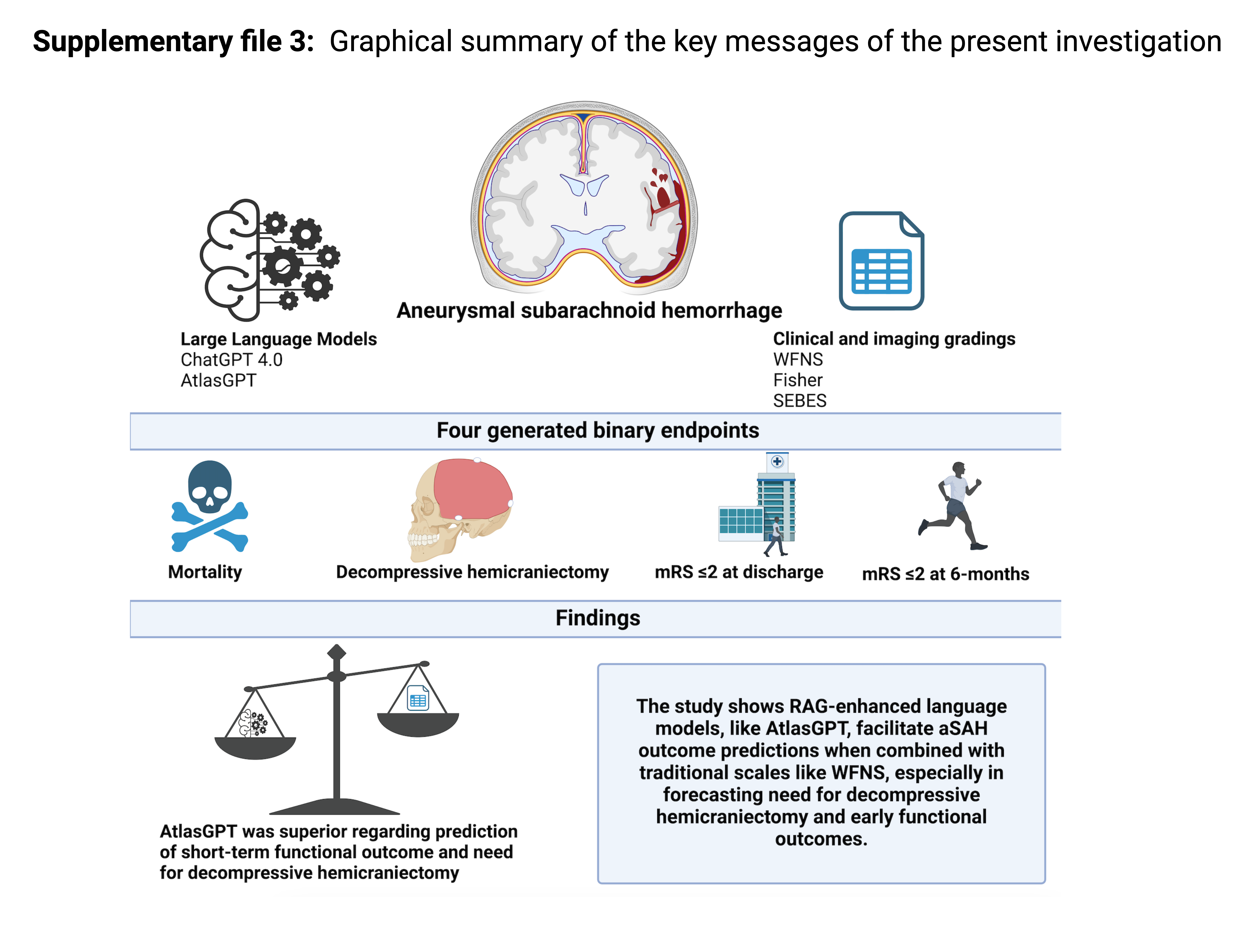

Supplement: Supplementary file 3 — Supplementary Material 3 [file 10143_2025_3194_MOESM3_ESM.png]
