# Supplementary material for: Beyond traditional prognostics: integrating RAG-enhanced AtlasGPT and ChatGPT 4.0 into aneurysmal subarachnoid hemorrhage outcome prediction
Source: Neurosurg Rev. 2025 Jan 11;48(1):40. doi: 10.1007/s10143-025-03194-w (PMC11723888; doi:10.1007/s10143-025-03194-w)
Supplement: Supplementary file 4 — Supplementary Material 4 [file 10143_2025_3194_MOESM4_ESM.docx]

| **Supplementary table 1.** Outcome characteristics | |
| --- | --- |
| In-hospital mortality | 18 (22.0%) |
| Decompressive hemicraniectomy during treatment | 28 (34.1%) |
| Favorable outcome at discharge (mRS ≤2) | 23 (28.0%) |
| Favorable outcome at 6-months after aSAH (mRS ≤2)(excluding dead patients) | 30 (46.9%) |
| mRS = modified rankin scale |  |

| **Supplementary table 2.** Outcome analysis | | | | | | |
| --- | --- | --- | --- | --- | --- | --- |
| Prognosis | Metrics | AtlasGPT | ChatGPT 4.0 | WFNS | Fisher | SEBES |
| In-hospital mortality | Sensitivity | 54.7% | 50.0% | 59.4% | 26.2% | 39.0% |
|  | Specificity | 83.3% | 83.3% | 83.3% | 83.3% | 70.6% |
|  | Positive predictive value | 92.1% | 91.4% | 92.7% | 85.0% | 79.2% |
|  | Negative predictive value | 34.1% | 31.9% | 36.6% | 24.2% | 25.9% |
|  | AUC (95% CI) | 0.70 (0.57-0.83) | 0.67 (0.53-0.80) | 0.72 (0.60-0.84) | 0.54 (0.40-0.69) | 0.53 (0.38-0.68) |
|  | Youden´s Index | 0.38 | 0.33 | 0.43 | 0.1 | 0.1 |
|  | | | | | | |
| Need for decompressive hemicraniectomy | Sensitivity | 82.1% | 92.9% | 89.3% | 89.3% | 63.0% |
|  | Specificity | 77.8% | 63.0% | 53.7% | 30.9% | 77.6% |
|  | Positive predictive value | 65.7% | 56.6% | 50.0% | 40.3% | 63.0% |
|  | Negative predictive value | 89.4% | 94.4% | 90.6% | 85.0% | 79.2% |
|  | AUC (95% CI) | 0.80 (0.70-0.91) | 0.78 (0.68-0.88) | 0.76 (0.59-0.83) | 0.61 (0.48-0.73) | 0.71 (0.59-0.83) |
|  | Youden´s Index | 0.6 | 0.56 | 0.43 | 0.2 | 0.41 |
|  | | | | | | |
| Favorable outcome at discharge (mRS ≤2 | Sensitivity | 69.6% | 65.2% | 69.6% | 41.7% | 73.7% |
|  | Specificity | 79.7% | 79.3% | 72.9% | 83.1% | 40.4% |
|  | Positive predictive value | 57.1% | 55.6% | 41.5% | 50.0% | 17.9% |
|  | Negative predictive value | 87.0% | 85.5% | 85.4% | 79.0% | 72.3% |
|  | AUC (95% CI) | 0.75 (0.62-0.87) | 0.72 (0.59-0.85) | 0.74 (0.61-0.87) | 0.62 (0.48-0.76) | 0.53 (0.38-0.67) |
|  | Youden´s Index | 0.49 | 0.45 | 0.43 | 0.25 | 0.14 |
|  | | | | | | |
| Favorable outcome at 6-months after aSAH (mRS ≤2) | Sensitivity | 60.0% | 60.0% | 76.7% | 32.3% | 66.7% |
|  | Specificity | 81.2% | 78.1% | 71.9% | 84.4% | 50.0% |
|  | Positive predictive value | 75.0% | 72.0% | 71.9% | 66.7% | 50.0% |
|  | Negative predictive value | 68.4% | 67.6% | 76.7% | 57.4% | 66.7% |
|  | AUC (95% CI) | 0.69 (0.56-0.83) | 0.69 (0.56-0.83) | 0.76 (0.64-0.88) | 0.58 (0.44-0.72) | 0.58 (0.43-0.73) |
|  | Youden´s Index | 0.41 | 0.38 | 0.49 | 0.14 | 0.17 |

**Supplementary Table 3**

| **Z-Test Comparing the need for decompressive hemicraniectomy in aSAH at admission** | | | |
| --- | --- | --- | --- |
| **Comparison** | **AUC Difference** | **Z-Score** | **Interpretation** |
| **AtlasGPT vs. ChatGPT 4.0** | 0.02 | 0.27 | Not statistically significant |
| **AtlasGPT vs. WFNS** | 0.04 | 0.54 | Not statistically significant |
| **AtlasGPT vs. SEBES** | 0.09 | 1.11 | Not statistically significant |
| **AtlasGPT vs. FISHER** | 0.19 | 2.29 | Statistically significant (p < 0.05) |
| **ChatGPT 4.0 vs. WFNS** | 0.02 | 0.28 | Not statistically significant |
| **ChatGPT 4.0 vs. SEBES** | 0.07 | 0.89 | Not statistically significant |
| **ChatGPT 4.0 vs. FISHER** | 0.17 | 2.10 | Statistically significant (p < 0.05) |
| **WFNS vs. SEBES** | 0.05 | 0.63 | Not statistically significant |
| **WFNS vs. FISHER** | 0.15 | 1.85 | Not statistically significant |
| **SEBES vs. FISHER** | 0.10 | 1.14 | Not statistically significant |

| **Z-Test Comparing the need for 30-d in-hospital survival after aSAH** | | | |
| --- | --- | --- | --- |
| **Comparison** | **AUC Difference** | **Z-Score** | **Interpretation** |
| **AtlasGPT vs. ChatGPT 4.0** | 0.03 | 0.35 | Not statistically significant |
| **AtlasGPT vs. WFNS** | 0.10 | 1.17 | Not statistically significant |
| **AtlasGPT vs. SEBES** | 0.17 | 1.97 | Statistically significant (p < 0.05) |
| **AtlasGPT vs. FISHER** | 0.16 | 1.88 | Not statistically significant |
| **ChatGPT 4.0 vs. WFNS** | 0.07 | 0.82 | Not statistically significant |
| **ChatGPT 4.0 vs. SEBES** | 0.14 | 1.63 | Not statistically significant |
| **ChatGPT 4.0 vs. FISHER** | 0.13 | 1.55 | Not statistically significant |
| **WFNS vs. SEBES** | 0.07 | 0.85 | Not statistically significant |
| **WFNS vs. FISHER** | 0.06 | 0.73 | Not statistically significant |
| **SEBES vs. FISHER** | 0.01 | 0.12 | Not statistically significant |

| **Z-Test Comparing the prediction of good functional outcome (mRS≤2) at discharge** | | | |
| --- | --- | --- | --- |
| **Comparison** | **AUC Difference** | **Z-Score** | **Interpretation** |
| **AtlasGPT vs. ChatGPT 4.0** | 0.02 | 0.26 | Not statistically significant |
| **AtlasGPT vs. WFNS** | 0.04 | 0.49 | Not statistically significant |
| **AtlasGPT vs. SEBES** | 0.09 | 1.11 | Not statistically significant |
| **AtlasGPT vs. FISHER** | 0.19 | 2.30 | Statistically significant (p < 0.05) |
| **ChatGPT 4.0 vs. WFNS** | 0.02 | 0.23 | Not statistically significant |
| **ChatGPT 4.0 vs. SEBES** | 0.07 | 0.84 | Not statistically significant |
| **ChatGPT 4.0 vs. FISHER** | 0.17 | 2.05 | Statistically significant (p < 0.05) |
| **WFNS vs. SEBES** | 0.05 | 0.61 | Not statistically significant |
| **WFNS vs. FISHER** | 0.15 | 1.83 | Not statistically significant |
| **SEBES vs. FISHER** | 0.10 | 1.22 | Not statistically significant |

| **Z-Test Comparing the prediction of good functional outcome (mRS≤2) at 6-months after aSAH** | | | |
| --- | --- | --- | --- |
| **Comparison** | **AUC Difference** | **Z-Score** | **Interpretation** |
| **AtlasGPT vs. ChatGPT 4.0** | 0.00 | 0.00 | Not statistically significant |
| **AtlasGPT vs. WFNS** | 0.07 | 0.77 | Not statistically significant |
| **AtlasGPT vs. SEBES** | 0.11 | 1.20 | Not statistically significant |
| **AtlasGPT vs. FISHER** | 0.11 | 1.20 | Not statistically significant |
| **ChatGPT 4.0 vs. WFNS** | 0.07 | 0.77 | Not statistically significant |
| **ChatGPT 4.0 vs. SEBES** | 0.11 | 1.20 | Not statistically significant |
| **ChatGPT 4.0 vs. FISHER** | 0.11 | 1.20 | Not statistically significant |
| **WFNS vs. SEBES** | 0.18 | 2.00 | Statistically significant (p < 0.05) |
| **WFNS vs. FISHER** | 0.18 | 2.00 | Statistically significant (p < 0.05) |
| **SEBES vs. FISHER** | 0.00 | 0.00 | Not statistically significant |
